# Unexpected Results from Taking Drugs

**Published:** 1902-10-25

**Authors:** 


### UNEXPECTED RESULTS FROM TAKING DRUGS.

Sir Lauder Brunton [1] has recently related some curious and unexpected results which have sometimes followed the administration of drugs. I remember, he said, reading the case of a child who was said to have died from one drop of laudanum, but this laudanum had been kept on a mantelshelf for a considerable time and the mouth of the bottle only stopped by a twisted piece of paper, so that the original tincture of opium had become converted into a strong liquid extract by evaporation. Here was a case in which by keeping, and in a sense deteriorating, the drug had become far stronger than before. But occasionally the very purity of drugs may alter their effect for the worse. Professor Leech pointed out that artificial sodium salicylate owes its inferiority to natural salicylate, not to the presence of any impurity, but really to the absence of a certain amount of methyl salicylate which exists in the natural product.

Some time ago, on prescribing potassium nitrate with the view of lessening high arterial tension and arresting epistaxis, Sir Lauder Brunton found that the patient was immediately relieved by the use of saltpetre which he got at an oil shop, but on getting the prescription made up at a chemist's, with pure potassium nitrate, the epistaxis began again. On hearing of this, he at once suspected that the ordinary saltpetre contained a small amount of nitrite, and on adding about half a grain of sodium nitrite to the 15 or 20 grains of potassium nitrate that he had been taking the epistaxis ceased immediately. This result explained an observation which had been made by a very old doctor, who had told him that although he belonged to a very gouty family he kept gout away by taking 20 grains of nitrate along with 15 or 20 grains of potassium bicarbonate in a tumbler of water every morning, and—here was the point—that the nitre he got from a gunmaker was always better than what he got from a chemist. This was probably due to a small admixture of nitrite which tended to keep down the high tension which his gouty kidneys would otherwise have produced.

Quinine may act very differently according to the amount of acid in the stomach. In tropical countries quinine is frequently swallowed by the teaspoonful, and a great part of this is often wasted because there is an insufficient amount of acid in the intestinal canal to dissolve it. Should, however, a patient take quinine in this way and have several lemon squashes immediately afterwards, so much quinine may be dissolved by the citric acid they contain as to give rise to unpleasant effects. Calomel again may vary unexpectedly in its effects. In persons who live upon a vegetable diet and are accustomed to take but little salt, calomel appears only to have a slight action, but in those who take a lot of salt, or are accustomed to live upon salt provisions, a larger quantity of calomel is converted into corrosive sublimate, and thus an unexpectedly violent action may be produced. Sulphide of antimony, on the other hand, is dissolved by alkalies, so that when compound calomel pills, which contains this drug, is given along with alkalies, a degree of gastro-intestinal irritation may be produced which does not occur in other conditions. The solubility of certain resinous purgatives such as aloes, scammony, jalap and podophyllin is also much increased by alkalies, and some of the unexpected excess or lack of action of these drugs which occasionally manifests itself is doubtless due not to impurity of the drug but to the amount of alkali present at the time in the intestinal canal.

[1] Brit. Med. Jour., Oct. 11.

